# pH-Sensitive Multiliposomal Containers for Encapsulation and Rapid Release of Bioactive Substances

**DOI:** 10.3390/molecules30122608

**Published:** 2025-06-16

**Authors:** Anna A. Efimova, Tatyana A. Abramova, Igor V. Yatsenko, Alexey V. Kazantsev, Denis V. Pozdyshev, Nikolay V. Lukashev, Vladimir I. Muronets, Alexander A. Yaroslavov

**Affiliations:** 1 Department of Chemistry, M.V. Lomonosov Moscow State University, Leninskie Gory 1-3, 119991 Moscow, Russiaigor.yatsenko.2004@mail.ru (I.V.Y.);; 2 Belozersky Institute of Physico-Chemical Biology, M.V. Lomonosov Moscow State University, Leninskie Gory 1/40, 119992 Moscow, Russia

**Keywords:** liposome, nanocontainer, pH-sensitive, polyethylene glycol, molecular switch, bioactive compound, multiliposomal, cytotoxicity, controlled drug delivery

## Abstract

A new method of the design of stimuli-sensitive multiliposomal containers for encapsulation and controlled drug release is described. Despite quite a wide choice of pH-sensitive containers, there is still a considerable challenge to synthesize those that respond quickly to small variations in pH and release most of the encapsulated drug in a short time. The suggested AMS-containing multiliposomal complexes demonstrated an excellent rate of encapsulated substance release under altering the pH of the outer solution. To improve the efficiency of the delivery of bioactive compounds to target cells and to increase the therapeutic effect, pH-sensitive liposomes were concentrated on the surface of the carrier- PEG-coated cationic liposomes. A pH-sensitive ampholytic derivative of cholan-24-oic acid embedded into the membrane of anionic liposomes allowed the rapid release of the cargo in the areas of low pH, such as tumors, inflammation sites, etc. The diameter of the complexes was optimized for passive targeting and typically ranged from 250 to 400 nm. The biodegradability of liposomes ensured enzymatic destruction of the multiliposomal containers and their elimination from the body after performing their transport function. The multiliposomal complexes and products of their biodegradation demonstrated low cytotoxicity. The composition of multiliposomal complexes, in particular, the amount of PEGylated lipid in the bilayer, was estimated to provide a high speed of the cargo release upon changing the pH. The novel developed pH-sensitive containers show potential for biomedical applications.

## 1. Introduction

**Figure 1:**
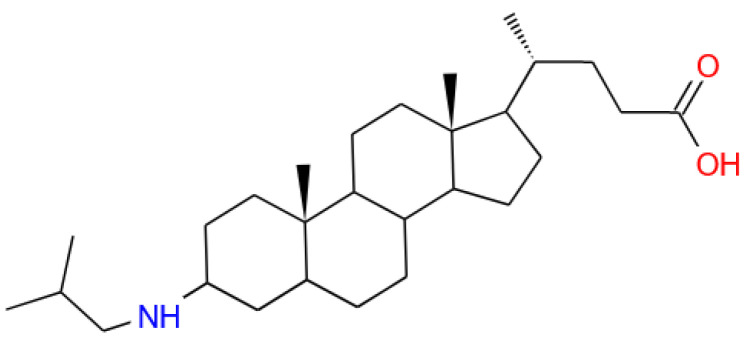
3-(isobutylamino)cholan-24-oic acid, AMS.

Modern therapy for serious diseases is often accompanied by significant adverse effects. Therefore, the development of new approaches and effective treatment methods remains an urgent task in biomedicine [1,2,3]. In some cases, biologically active substances are immobilized in bilayer lipid vesicles (liposomes) capable of releasing a drug under the action of an external stimulus [4,5,6,7]. Recent studies have shown that zwitterionic liposomes exhibit promising results in the release of bioactive substances. A stimuli-responsive liposomal system was designed to control the delivery of curcumin using enzyme-specific and hyperthermic stimuli [8]. In this case, curcumin release was initiated in response to phospholipase A2, which degraded phospholipids, enabling a highly targeted effect. Another example of a liposomal system providing effective nanomedicines for efficient tumor entrance is a tertiary amine oxide (TAO)-containing zwitterionic liposomal nanocarrier that hitchhikes red blood cells (RBCs) to tumor blood vessels and enters solid tumors through transcytosis [9]. The use of liposomes with an embedded pH-sensitive molecular switch [10,11] allows the release of encapsulated drug after the container enters areas with a lower pH value, for example, in tumors, inflammation sites, or lysosomes [12]. The pH for pathological zones was established to vary from 5.9 to 6.9 [13,14]. Recently, we have suggested a new type of ampholytic molecular switch (AMS, Figure 1.), the lithocholic acid derivative with cationic and anionic groups attached to the opposite parts of the steroid core [15]. The AMS reorients within the lipid bilayer in response to external pH variations [16]. These structural transitions trigger extensive reorganization of hydrophobic domains, destabilizing the membrane, generating transient defects, and enabling rapid cargo release. AMS-containing liposomes demonstrated the rapid release of the cargo initiated by acidification [17].

An increase in the efficiency of drug delivery can be achieved by binding the liposomal containers on the surface of colloidal particles [18,19]. The concentration of liposomes loaded with bioactive substances within a rather small volume could lead to an increase in the therapeutic effect of the drug. Anionic liposomes can be electrostatically adsorbed on the surface of cationic colloidal particles, which is expected to be accompanied by enhanced action of the encapsulated active ingredient. Moreover, this is also the way to obtain multifunctional medical compositions capable of carrying a mixture of different drugs in a required ratio. We have recently demonstrated the possibility of constructing multiliposomal conjugates by means of electrostatic interactions of small negatively charged liposomes with larger positively charged liposomes [20]. The complexes containing several tens of negatively charged liposomes per cationic liposome displayed no cytotoxicity and were capable of enzyme-induced degradation down to nanometer particles.

In this work, we have combined the two approaches described above and created pH-sensitive multiliposomal constructs (MLC) by electrostatic adsorption of anionic pH-sensitive liposomes on the surface of the cationic carrier liposomes.

To prevent the fusion of lipid membranes upon contact with each other, followed by the premature release of the cargo, the cationic liposomes have to be modified with a hydrophilic polymer, polyethylene glycol [21], that nowadays is most commonly used in biomedicine [22,23,24,25,26,27]. For this, PEG-phospholipids are incorporated into bilayer [28,29]. The presence of PEG-phospholipid in the lipid bilayer may interfere with the AMS functioning. Therefore, we tested whether the AMS would function effectively in PEG-containing multiliposomal complexes.

Here, we report how the amount of PEGylated lipids in the membrane of cationic liposomes affects the integrity of anionic liposomes adsorbed on their surface, as well as AMS functioning in response to acidification of the external solution. We also investigate the cytotoxicity of the formed pH-sensitive multiliposomal constructs and their biodegradation products.

## 2. Results and Discussion

### 2.1. Physicochemical Characteristics of Cationic and Anionic Liposomes

The liposomes were prepared according to the protocol described in the Section 3. The positively charged liposomes were formed from cationic 1,2-dioleoyl-3-trimethylammonium-propane (DOTAP^1+^), electroneutral dipalmitoylphosphatidylcholine (DPPC) and PEGylated lipid-1,2-dipalmitoyl-3-phosphoethanolamine-N-[methoxy(polyethylene glycol)-1000] (DPPE-PEG). The pH-sensitive anionic liposomes were obtained using negatively charged cardiolipin (CL^2−^), electroneutral dioleoylphosphatidylcholine (DOPC), and ampholytic molecular switch (AMS). AMS was a derivative of 3-(isobutylamino)cholan-24-oic acid containing anionic (carboxylic) and cationic (isobutylamino) groups attached to the ends of the steroid part (Figure 1). The chemical structures and compositions of cationic and anionic liposomes are presented in Supplementary Materials (Table S2).

Both types of lipid vesicles were examined via dynamic light scattering (DLS) and laser microelectrophoresis. The results are shown in Table 1. It was established that the liposomal size varied but always remained in 40 ± 10 nm intervals for negatively charged vesicles and 163 ± 20 nm for positively charged. As follows from Table 1, there are slight variations in the size of anionic liposomes due to the different amounts of PEGylated lipids. The size was also determined by the buffer in which the liposomes were prepared, as well as whether the liposomes were empty or contained an encapsulated substance. The size distribution of anionic and cationic vesicles and their saturated complexes are presented in Supplementary Materials (Figure S6).

### 2.2. Release of the Cargo by CL^2−^/DOPC/AMS Liposomes Under Changes in pH

It was previously established that pH-sensitive DOPC/AMS liposomes, prepared at basic pH, retain their integrity at pH 8.0, 7.5, and 7.0 but quickly release their cargo when the buffer is replaced with a more acidic one: 85–95% of the cargo outflowed from the vesicles in the first minutes of pH decreasing to 6.0 and 6.5 [15]. Our prior research established that this mechanism originates from pH-induced structural perturbations and changes in AMS location in the membrane, which induces temporal defect formation. This destabilization of the bilayer is followed by the release of encapsulated agents [16,17].

To allow pH-sensitive liposomes to electrostatically bind to the cationic carrier, negatively charged cardiolipin was incorporated into their membrane. In the first step, we tested whether changing the membrane composition would affect the efficiency of the switch functioning. In other words, we have checked whether CL^2−^/DOPC/AMS liposomes would behave similarly to DOPC/AMS liposomes or CL^2−^ incorporation would interfere with the switch acting. The integrity of pH-sensitive anionic liposomes was examined by fluorescence spectroscopy [18]. The inner capacity of CL^2−^/DOPC/AMS vesicles was loaded with a self-quenching concentration of the fluorescent probe carboxyfluorescein (CF). Compromised membrane integrity would be accompanied by dye outflow into the external environment, reducing CF concentration and increasing the suspension fluorescence. The full destruction of the vesicles was initiated by adding Triton X-100 and was followed by a three-fold fluorescence increase [18,30]. Membrane integrity of pH-sensitive liposomes in MLCs was assessed via fluorescence spectroscopy. CL^2−^/DOPC/AMS liposomes were loaded with self-quenched carboxyfluorescein (CF), where membrane disruption triggers CF dilution in the medium, reducing self-quenching and amplifying fluorescence intensity.

**Figure 2:**
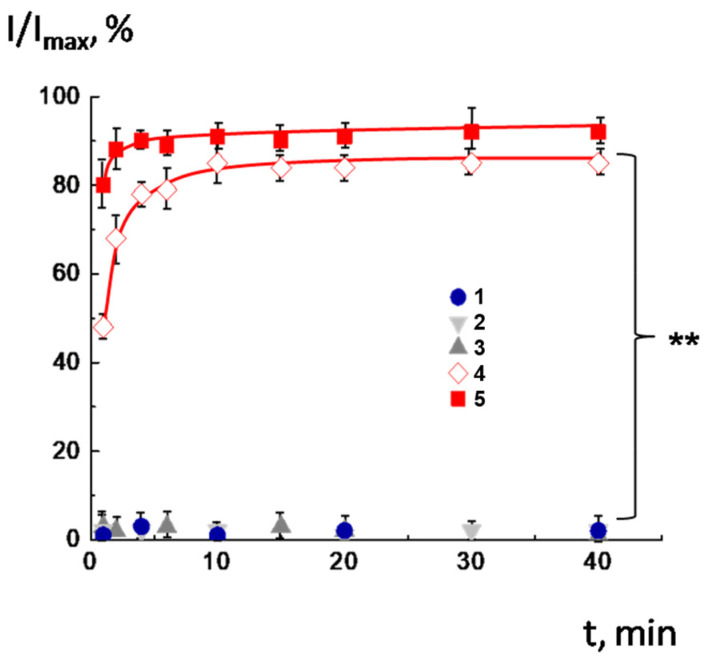
Time dependence of relative fluorescence of CF-loaded CL^2-^/DOPC/AMS vesicles at different pH values. Vesicles were prepared at pH = 8.0 (1) and then transferred into the buffer with pH = 7.5 (2), 7.0 (3), 6.5 (4), and 6.0 (5). Lipid concentration 1 mg/mL. The asterisk (**) annotates the curves which are identical with *p* < 0.001.

The CF-loaded CL^2−^/DOPC/AMS liposomes were prepared in a pH 8.0 solution, and then the pH was reduced to 7.5, 7.0, 6.5, and 6.0. The results of the experiment are shown in Figure 2. As follows from the figure, the liposomes maintained their integrity at a pH of 8.0 (curve 1) and preserved it after transferring to physiological pH ranges (7.0–7.5) (curves 2 and 3). The release of fluorescence dye began at pH = 6.5 and 6.0 (curves 4 and 5). Notably, carboxyfluorescein (CF) release kinetics was very effective, with 85–90% of encapsulated dye escaping vesicles within a few minutes after acidification. This observation highlights that the inclusion of cardiolipin in the membrane does not interfere with the switching mechanism. At pH = 8.0, the AMS was in anionic form; hence, it was vertically orientated: the negatively charged carboxyl group was located in the outer part of the bilayer. When protonated, the AMS is reoriented at the bilayer, such that the amino group is located in the outer region. Such transfer of the anionic form to the cationic caused by pH alteration was followed by rearrangement of the AMS molecules in the lipid membrane and disordering of the hydrophobic part of the bilayer. Therefore, the temporal defects in the membrane were formed, resulting in a fast release of the cargo.

### 2.3. Complexation of pH-Sensitive Anionic Liposomes with Cationic Liposomes

In subsequent investigations, pH-responsive anionic vesicles were complexed with cationic ones. When creating bioactive substance delivery systems, it is important to prevent premature leakage of the cargo by preserving the integrity of containers and ensuring controlled therapeutic release. We found out earlier that the incorporation of PEGylated lipid into the membrane of cationic liposomes preserves the integrity of anionic liposomes at multiliposomal constructs owing to a mild hydrophilic layer formed by PEG between oppositely charged vesicles [20]. Taking this into account, we prepared core cationic liposomes with embedded PEGylated lipids: the molar ratio of PEGylated lipids varied from 0.05 to 0.2.

**Figure 3:**
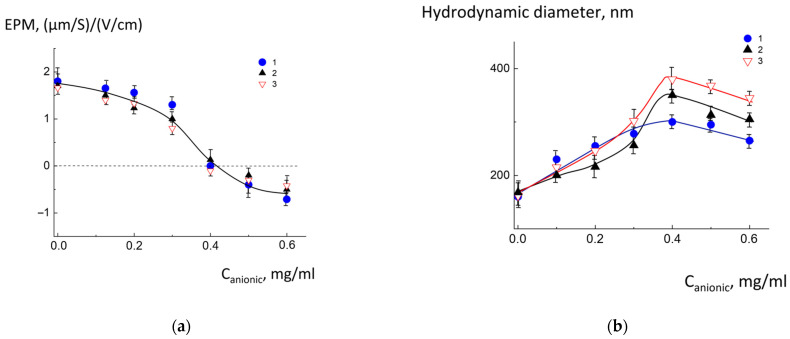
Dependence of electrophoretic mobility (**a**) and hydrodynamic diameter (**b**) of the complexes of anionic CL^2−^/DOPC/AMS vesicles with cationic DOTAP^1+^/DPPC/DPPE-PEG vesicles on total anionic liposome concentration C_anionic_. Molar ratio of DPPE-PEG 0.05 (1), 0.1 (2) and 0.2 (3). Total cationic liposome concentration C_cationic_ 1 mg/mL. 10^−2^ M TRIS buffer, pH = 8.0.

Electrostatic assembly of anionic CL^2−^/DOPC/AMS and cationic DOTAP^1+^/DPPC/DPPE-PEG liposomes was conducted in TRIS buffer (pH 8.0). EPM analysis via laser microelectrophoresis demonstrated charge neutralization across all cationic liposome types, confirming the formation of electrostatic complexes with anionic liposomes adsorbed onto cationic cores. The results are presented in Figure 3.

As follows from Figure 3a (curves 1–3), for all types of investigated liposomes, the electroneutral complexes (EPM = 0 (μm/s)/(V/cm)) were obtained at the C_anionic_ = 0.4 mg/mL. This definitely indicates that for all liposomal compositions, the same cardiolipin amount participated in the formation of an electrostatic complex. The charge of the complexes became negative with increasing concentration of anionic liposomes.

The multiliposomal aggregates formation was characterized by particle size augmentation and charge neutralization, driven by adsorption-mediated interactions between cationic and anionic groups of the vesicles (Figure 3b). The maximum hydrodynamic diameter was observed at EPM = 0 (μm/s)/(V/cm). Further increase of C_anionic_ resulted in a slight decrease in size due to the negative charge caused by excess of anionic liposomes. At saturation, the diameter of the complexes was 340 ± 40 nm. Nanoparticle sizes optimized for passive targeting in drug delivery systems typically range from 200 to 400 nm [31,32,33]. The obtained multiliposomal complexes meet this condition and, therefore, have potential as drug carriers.

It was established that the quantitative binding of anionic liposomes was observed up to C_anionic_ = 0.6 mg/mL. Until this concentration, all added CL^2−^/DOPC/AMS liposomes were complexed with the cationic, and no free anionic liposomes appeared in the suspension. The details of the separate experiment are presented at Supplementary Materials, Procedure S2.

The liposomal aggregates demonstrated colloidal stability in physiological salt concentrations (0.15–0.2 M NaCl), with no measurable particle size reduction observed under these conditions. However, increased ionic strength (0.3 M NaCl) caused dissociation of the complexes, returning the particle size to 163 ± 20 nm, a value corresponding to cationic liposomes before complexation. This response to the salt addition confirmed the electrostatic nature of the vesicle interaction.

Particle behavior over an extended period was also estimated: at 4 °C, the multiliposomal complexes retain their size for at least 7 days. The data on the hydrodynamic diameter of the complexes are presented in SM (Table S2). It was also shown that multiliposomal complexes are stable under physiological conditions: at a temperature of 37 °C in a saltwater solution with a NaCl concentration of 0.15–0.2 M, as well as in incubation media RPMI 1640 and DMEM/F12, the size of the MLC was preserved for 7 days of observation (Supplementary Materials, Table S3).

### 2.4. Integrity of CL^2−^/DOPC/AMS Lipid Membranes in Multiliposomal Complexes

PEGylated lipid was incorporated into the membrane of the cationic liposomes to prevent uncontrolled premature release of the cargo because of the fusion of membranes upon contact with each other. At the same time, the presence of PEG-phospholipid may also interfere with the AMS functioning. Therefore, the next step was to estimate the membrane composition that ensures the preservation of the integrity of anionic liposomes under initial conditions but, at the same time, does not interfere with the AMS disordering action when the buffer is replaced with a more acidic one.

**Figure 4:**
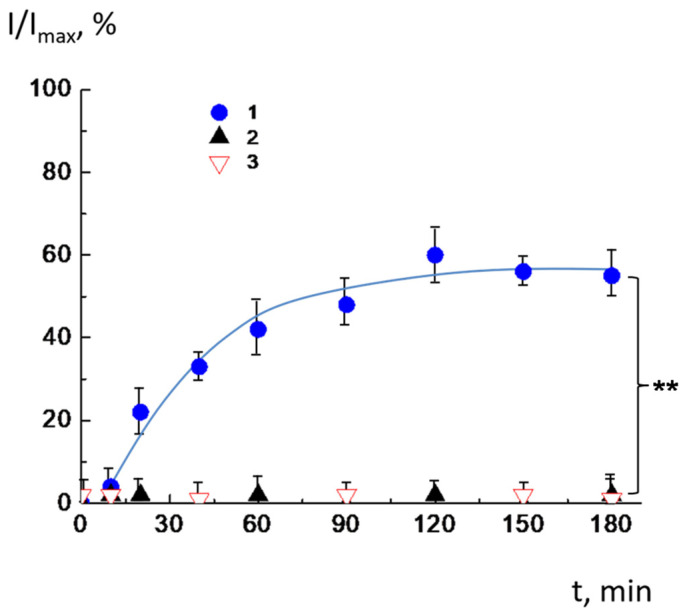
Time dependence of relative fluorescence of complexes of CF-loaded anionic CL^2−^/DOPC/AMS vesicles with cationic DOTAP^1+^/DPPC/DPPE-PEG vesicles. Molar ratio of DPPE-PEG 0.05 (1), 0.1 (2) and 0.2 (3). The total liposome concentration: C_cationic_ = 1 mg/mL, C_anionic_ =0.4 mg/mL. 10^−2^ M TRIS buffer, pH = 8.0. The asterisk (**) annotates the curves which are identical with *p* < 0.01.

The following experiment was carried out to determine the amount of incorporated PEGylated lipid that is sufficient to form a protective layer. We prepared cationic liposomes with different ratios of PEGylated lipids, mixed them with anionic pH-sensitive liposomes loaded with CF, and monitored the integrity of anionic membranes after complex formation. The experiment was performed at pH = 8. The membrane integrity of pH-sensitive liposomes in complexes was examined by measuring the fluorescence of CF-loaded liposomes as described above. The results of the experiment are presented in Figure 4. The CL^2−^/DOPC/AMS vesicles lost integrity when interacting with positively charged ones with the ratio of PEGylated lipids equal to 0.05 (Figure 4, curve 1). In this case, the defect formation in the vesicle membrane was followed by the outflow of incorporated dye. Obviously, the percentage of PEGylated lipid was inappropriate to generate a protective hydrophilic layer and to completely block the contacts of oppositely charged bilayers. Withal, for complexes with a molar content of PEGylated lipid of 0.1 and 0.2, no increase in fluorescence was detected (Figure 4, curves 2, 3). Thus, for these bilayer compositions, the intact integrity of anionic vesicles in complexes with cationic vesicles is observed. So, cationic liposomes with PEGylated lipid molar content equal to 0.1 and 0.2 were chosen for further investigation.

**Figure 5:**
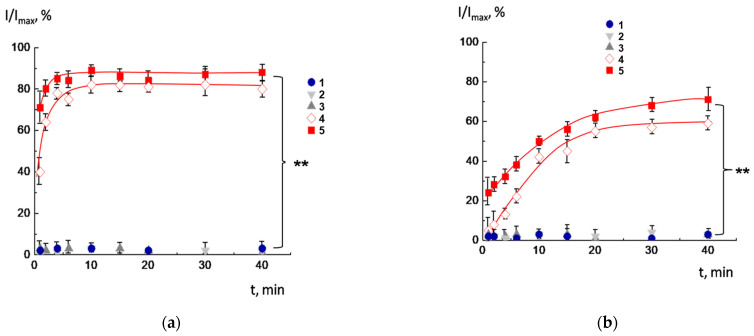
Time dependence of relative fluorescence of complexes of CF-loaded anionic CL^2-^/DOPC/AMS vesicles with cationic DOTAP^1+^/DPPC/DPPE-PEG vesicles at different pH values. The molar ratio of DPPE-PEG is equal to 0.1 (**a**) and 0.2 (**b**). Multiliposomal complexes were prepared at pH = 8.0 (1) and then transferred into the buffer with pH = 7.5 (2), 7.0 (3), 6.5 (4), and 6.0 (5). Total liposome concentration: C_cationic_ = 1 mg/mL, C_anionic_ =0.4 mg/mL. The asterisk (**) annotates the curves which are identical with *p* < 0.001.

Then, we tested whether the AMS would function effectively in PEG-containing multiliposomal complexes. We wanted to make sure that PEGylated lipids did not interfere with the rapid release of the encapsulated substance in response to acidification. For this, saturated multiliposomal complexes of CF-loaded anionic CL^2−^/DOPC/AMS liposomes with cationic DOTAP^1+^/DPPC/DPPE-PEG liposomes were prepared in a buffer solution with pH 8.0 and then transferred to buffer solutions with lower pH values. The experiment was carried out for two previously selected molar ratios of PEGylated lipids: 0.1 and 0.2. The results of the experiment are shown in Figure 5. It should be noted that AMS, being a part of a multiliposomal complex with a molar ratio of DPPE-PEG equal to 0.1 (Figure 5a), works almost as effectively as in individual liposomes (Figure 2). The liposomes retained their integrity at pH 8.0, 7.5, and 7.0 (curves 1–3), but 80–90% of dye was released from the liposomes within the first minutes after acidification (curves 4 and 5). However, for MLC with a ratio of PEGylated lipid of 0.2, the switch action was not effective (Figure 5b). Liposomes released only 20–30% in the first 2–5 min after decreasing pH down to 6.0 and 6.5. Over time, the amount of released dye increased, but even after 40 min, it did not exceed 55–70%. Apparently, in this case, the PEG shell of the cationic liposomes prevented the instantaneous release from the anionic liposomes.

Thus, cationic liposomes containing 0.1 PEGylated lipid have the greatest potential as a carrier for pH-sensitive anionic liposomes. Therefore, the cationic liposomes of this composition were selected to form multiliposomal complexes for further experiments.

**Figure 6:**
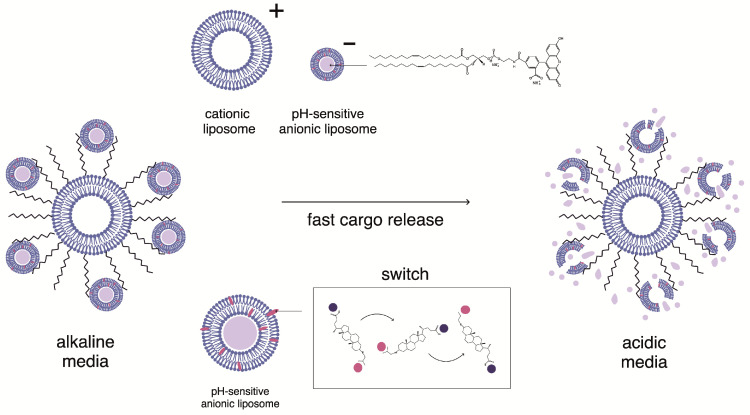
Schematic illustration of pH-sensitive multiliposomal complex.

A schematic illustration of a pH-sensitive multiliposomal complex is presented in Figure 6.

### 2.5. Biodegradation of Multiliposomal Complexes

**Figure 7:**
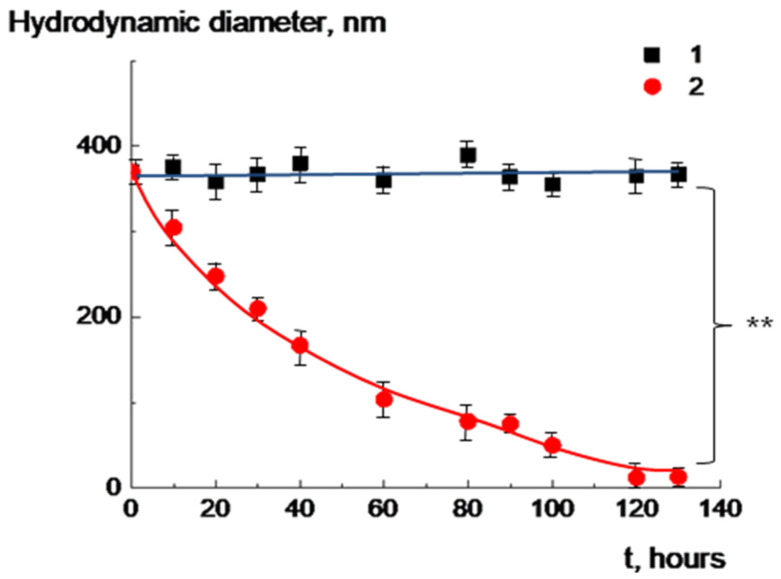
Time-dependent change in the size of complex of anionic CL^2−^/DOPC/AMSvesicles with cationic DOTAP^1+^/DPPC/DPPE-PEG vesicles in the absence (1) and in the presence of lipase (2). Saturated multiliposomal complexes were prepared at pH = 8.0 and transferred to pH = 7.5 before lipase addition. The molar ratio of DPPE-PEG is equal to 0.1. Total liposome concentration: C_cationic_ = 1 mg/mL, C_anionic_ =0.4 mg/mL. Lipase concentration 5 × 10^−1^ mg/mL. The asterisk (**) annotates the curves which are identical with *p* < 0.001.

Previous studies have shown that multicomponent liposomal systems undergo enzymatic degradation with esterase-mediated cleavage of lipid ester bonds [18,19,34]. In accordance with prior observations, the addition of lipase was accompanied by hydrolysis of ester bonds and a gradual decrease in particle size: after 120 h, the size reached nanoscale dimensions (Figure 7). Particles of 10–15 nm can be easily removed from the body [35]. In the control experiment, no change in the size of the complex was observed in the absence of an enzyme. The light scattering results were confirmed by means of TEM (Supplementary Materials, Figure S7).

### 2.6. Cytotoxicity of pH-Sensitive Multiliposomal Complexes and the Products of Their Biodegradation

Finally, the cytotoxicity of the individual liposomes and the saturated multiliposomal complex of the optimal lipid composition (with the molar ratio of DPPE-PEG equal to 0.1) and products of their enzymatic degradation was quantified at MCF-7 breast carcinoma cells using a conventional MTT assay, as described in Supplementary Materials (Procedure S4).

**Figure 8:**
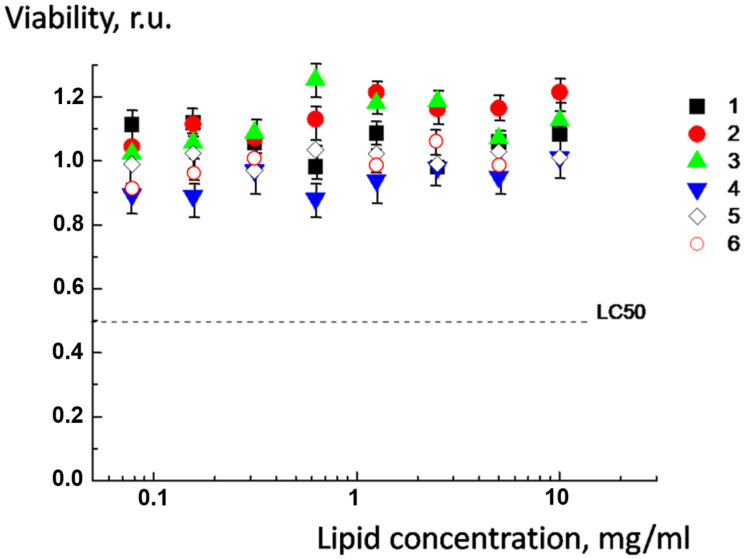
Viability of MCF-7 cells vs. lipid concentration before (1–3) and after (4–6) addition of lipase. Anionic CL^2−^/DOPC/AMS vesicles (1); cationic DOTAP^1+^/DPPC/DPPE-PEG vesicles (2); saturated multiliposomal complex (3). Time after lipase addition: 0.5 (4), 36 (5) and 72 h (6). The molar ratio of DPPE-PEG is equal to 0.1. Saturated multiliposomal complexes were prepared at pH = 8.0. Total lipid concentration used for liposome preparation: C_cationic_ = 10 mg/mL, C_anionic_ =4 mg/mL. Lipase concentration 5 × 10^−1^ mg/mL.

Cytotoxicity was evaluated via LC50 (concentration inducing 50% cell death) [30,36,37,38]. As follows from Figure 8, pH-sensitive anionic and cationic liposomes (curves 1 and 2), as well as multiliposomal construct (curve 3), were non-toxic; LC50 was not reached within the full interval of concentrations used in this experiment. The toxicity of cationic liposomes is determined by the charge density on the membrane surface. In this case, the positive charge density was low, and cationic liposomes did not demonstrate cytotoxicity up to a concentration of 10 mg/mL (curve 2). In the multiliposomal complex, the positive charge of the cationic liposomes was almost completely neutralized by the negative charge of the pH-sensitive liposomes (curve 3). The maximum lipid concentration achieved in this experiment was equal to 10 mg/mL.

Afterward, we added lipase to the complexes to initiate the degradation of the multiliposomal construction. We obtained the bio-destruction products after 30 min, one day, and three days, and each sample was tested for cytotoxicity. The resulting curves 4–6 for the viability of MCF-7 cells in the presence of hydrolyzed complexes are presented in Figure 8. For all samples, LC50 remained unattained, indicating a low cytotoxicity of the products at all stages of the degradation process.

Similar results were obtained on other cell lines: no cytotoxicity of the initial complexes and products of their biodegradation were determined on fibroblast cells WI-38 and Caco-2 cells, as well as mel P и Malme-3M.

## 3. Materials and Methods

### 3.1. Chemicals

1,2-Dioleoyl-sn-glycero-3-phosphocholine (DOPC), 2-dipalmitoyl-sn-glycero-3-phosphocholine (DPPC), diphosphatidyl glycerol (cardiolipin, CL^2−^), 1,2-dioleoyl-3-trimethylammonium-propane (DOTAP^1+^), 1,2-dipalmitoyl-sn-glycero-3-phosphoethanolamine-N-[methoxy-(polyethylene glycol)-1000] (ammonium salt) (DPPE-PEG), (all lipids are from Avanti, Tonawanda, NY, USA) and lipase (porcine pancreas, Sigma) were used without additional purification. The derivative of lithocholic acid, 3-(isobutylamino)cholan-24-oic acid (AMS), was synthesized according to Procedure S1, Figures S2–S5 (Supplementary Materials). The structural formulas are demonstrated in Table S2 (Supplementary Materials).

Tris(hydroxymethyl)aminomethane NH_2_C(CH_2_OH)_3_, fused sodium acetate CH_3_COONa (*both fresh grade*), sodium hydrogen phosphate dodecahydrate Na_2_HPO_4_·12H_2_O, sodium dihydrogen phosphate NaH_2_PO_4_·2H_2_O (*all chemically pure grade*) were used as received, and 10^−2^ M buffer solutions were prepared by weighing. Concentrated HCl, carboxyfluorescein (CF), and sodium chloride (*all chemically pure grade*) were also used as received.

Ultrapure water was generated through sequential purification steps: initial double distillation followed by advanced filtration via a Milli-Q system (Millipore, Burlington, MA, USA), which incorporated ion-exchange adsorption columns for organic contaminant removal and microporous filters to eliminate macroscopic particulates. The electrical conductivity of the final purified water was 0.8 μS/cm, confirming high-grade purity.

### 3.2. General Procedures

#### 3.2.1. Synthesis of pH-Sensitive Anionic Liposomes

Lipid vesicles were synthesized through the mixing of methanol–chloroform (1:3) solutions of lipid components and AMS, followed by controlled organic solvent evaporation under vacuum at 40 °C and a rotation speed of 60 rpm, dispersion of the formed film in 1 mL of 10^−2^ M buffer (pH 8.5) and further ultrasonic treatment [39,40] of the resulting mixture using 500-watt Cole-Parmer 4710 ultrasonic homogenizer at frequency of 22 kHz for 2 × 300 s at 40 °C. The obtained liposomes were centrifugated in a J-11 centrifuge (Beckman, Tokyo, Japan) for 4 min at 11,000 rpm to separate from titanium dust. The molar ratio of the anionic CL^2−^ headgroups ν_CL2−_ = [1/2CL^2−^]/([1/2CL^2−^] + [DOPC]) was equal to 0.1. The molar fraction of AMS was equal to 0.1. It was calculated as: ν_AMS_ = [AMS]/([AMS] + [DOPC] + [1/2CL^2−^]).

Negatively charged vesicles filled by carboxyfluorescein (fluorescent dye, CF) were synthesized as described above, but the lipid film was dispersed in a buffer containing 1 M CF. The liposomal suspension was dialized for 12 h against the buffer without CF, which was renewed every 1.5 h. For osmolarity balancing during dialysis, the outer buffer additionally contained 0.15 M NaCl.

The size of CL^2−^/DOPC/AMS liposomes was equal to 40 ± 10 nm.

#### 3.2.2. Synthesis of Cationic Liposomes

Vesicles were formed via a solvent evaporation–extrusion protocol. Lipid components were initially dissolved in a 1:3 methanol–chloroform mixture, followed by solvent removal under vacuum at 55 °C to form a lipid film. The resulting film was dispersed in 10^−2^ M buffer (pH 8.5), generating multilamellar vesicle suspension, which underwent 14 iterative extrusion cycles through a 200 nm pore-sized polycarbonate membrane using an Avanti Mini-Extruder (Avanti Research, Birmingham, AL, USA) to achieve size homogeneity. The final cationic liposome population exhibited a narrow size distribution of 163 ± 20 nm. The molar ratio of the cationic lipid DOTAP^1+^ ν_DOTAP1+_ = [DOTAP^1+^]/([DOTAP^1+^] + [DOPC]).

### 3.3. Methods

The hydrodynamic diameter of individual liposomes and multiliposomal complexes was determined by dynamic light scattering (DLS) in a temperature-controlled cell at a fixed scattering angle of 90° at Brookhaven Zeta Plus (Brookhaven Instruments, Nashua, NH, USA). The manufacturer-provided DynaLS software package (version 2.5) for size distribution analysis (cumulant method) was utilized for diameter calculations, with an error margin of ±7%. Diffusion coefficients were recalculated into mean hydrodynamic diameters with the Stokes−Einstein equation. Electrophoretic mobility measurements were performed by means of microelectrophoresis, using the same instrument and software, with an accuracy of ±3–5%.

The pH of solutions was established at Hanna 210 pH meter with an HI 1131B glass electrode, maintaining precision ±0.02 units.

Fluorescence intensity data were determined with an accuracy of ±5% by means of an F-4000 spectrofluorometer (Hitachi, Tokyo, Japan) in a temperature-controlled cell using 1 cm quartz cuvettes.

All experiments were carried out in quadruple replication.

Statistical treatment of experimental results was performed using one-way ANOVA. For the statistical comparison of two conditions, the Student’s *t*-test was used. Any *p*-value less than 0.01 was considered statistically significant and was denoted as **.

## 4. Conclusions

To conclude, pH-sensitive biodegradable nanocontainers of 250–400 nm size were obtained by complexation of anionic vesicles containing the derivative of cholan-24-oic acid with the cationic carrier liposomes. To prevent premature release of encapsulated substances as a result of possible fusion of lipid membranes, the cationic liposomes were modified with polyethylene glycol. It was found that the integrity of anionic liposomes depends on the PEGylated lipid ratio in the membrane of cationic carrier liposomes. The amount of PEGylated lipids also affects AMS functioning in response to acidification of the external solution. For the molar ratio of PEGylated lipid equal to 0.1, the integrity of AMS-containing anionic liposomes in complex with cationic is preserved, but at the same time, the anionic liposomes demonstrate excellent speed of the cargo release under changing pH. The multiliposomal complexes and products of their biodegradation demonstrated low cytotoxicity. The developed complexes show potential for nanopharmacology as effective delivery vehicles.

## Figures and Tables

**Table 1:** Hydrodynamic diameter, PDI, and electrophoretic mobility for liposomes of various compositions.

Composition of the Lipid Membrane	Hydrodynamic Diameter, nm	PDI	EPM, (μm/s)/(V/cm)
*Anionic liposomes* *			
CL^2−^/DOPC/AMS (1/8/1)	40 ± 10	0.17 ± 0.06	−2.6 ± 0.2
*Cationic liposomes* *			
DOTAP^1+^/DPPC/DPPE-PEG (1/8.5/0.5)	160 ± 17	0.11 ± 0.03	+1.8 ± 0.2
DOTAP^1+^/DPPC/DPPE-PEG (1/8/1)	162 ± 19	0.10 ± 0.04	+1.7 ± 0.2
DOTAP^1+^/DPPC/DPPE-PEG (1/7/2)	168 ± 15	0.12 ± 0.05	+1.7 ± 0.1

* 10^−2^ M TRIS buffer, pH = 8.0.

## Data Availability

The data are available from the corresponding author upon request.

## Supplementary Materials

The following supporting information can be downloaded at https://www.mdpi.com/article/10.3390/molecules30122608/s1. Table S1: Structure of compounds for liposome preparation; Procedure S1: AMS synthesis; Table S2: Hydrodynamic diameter of the MLC at 4 °C; Table S3: Hydrodynamic diameter of the multiliposomal complexes in 7 days of the incubation; Figure S1: Methyl 3β-(isobutylamino)-5β-cholan-24-oate (1H NMR, CDCl3); Figure S2: Methyl 3β-(isobutylamino)-5β-cholan-24-oate (13C NMR, CDCl3); Figure S3: 3β-(Isobutylamino)-5β-cholan-24-oic acid (1H NMR, CD3OD) (AMS); Figure S4: 3β-(Isobutylamino)-5β-cholan-24-oic acid (13C NMR, CD3OD) (AMS); Figure S5: Size distribution of anionic and cationic vesicles and their saturated complexes; Figure S6. TEM image of MLC. Figure S7. TEM image of the biodegradation products of MLC. Procedure S2: Degree of binding of pH-sensitive anionic liposomes with the cationic; Procedure S3: Evaluation of storage stability; Procedure S4: Evaluation of cytotoxicity.
