# Supplementary material for: pH-Sensitive Multiliposomal Containers for Encapsulation and Rapid Release of Bioactive Substances
Source: Molecules. 2025 Jun 16;30(12):2608. doi: 10.3390/molecules30122608 (PMC12195906; doi:10.3390/molecules30122608)
Supplement: Supplementary file 1 [file molecules-30-02608-s001.zip › molecules-3696344-supplementary.pdf]

Supplementary materials for article

pH-Sensitive Multiliposomal Containers for Encapsulation and Rapid Release of Bioactive Substances

Anna A. Efimova [1,*], Tatyana A. Abramova [1], Igor V. Yatsenko [1], Alexey V. Kazantsev [1], Denis V. Pozdyshev [2], Nikolay V. Lukashev [1], Vladimir I. Muronets [2] and Alexander A. Yaroslavov [1]

1 Department of Chemistry, M.V. Lomonosov Moscow State University, Leninskie Gory 1-3, 119991 Moscow, Russia
2 Belozersky Institute of Physico-Chemical Biology, M.V. Lomonosov Moscow State University, Leninskie Gory 1/40,119992 Moscow, Russia
* Correspondence: ephimova@belozersky.msu.ru; Tel.: +7-495-939-31-16

Table S1. Compounds for liposome preparation.

Lipid	Formula	Molar content in liposomes
DOPC	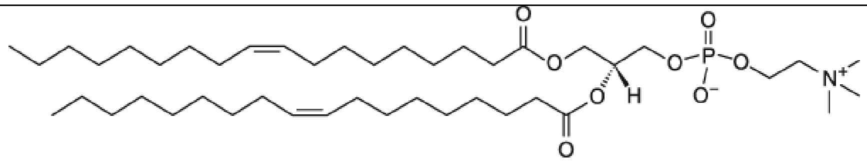	0.8
DPPC	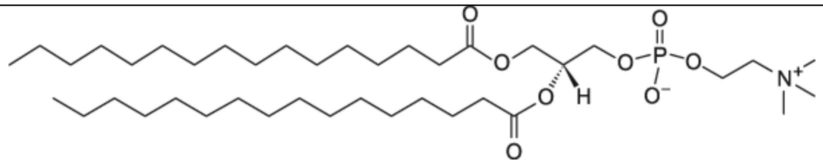	0.7-0.85
CL^{2-}	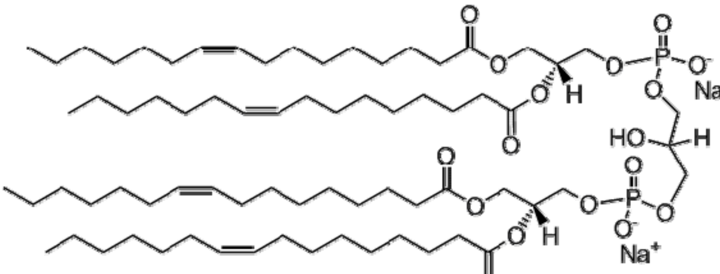	0.1*
$DOTAP^{1+}$	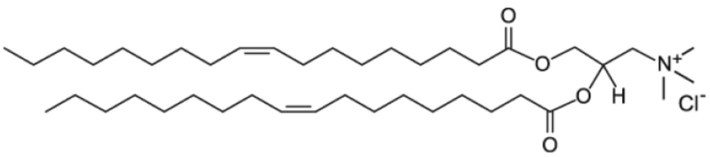	0.1**

DPPE-PEG1000	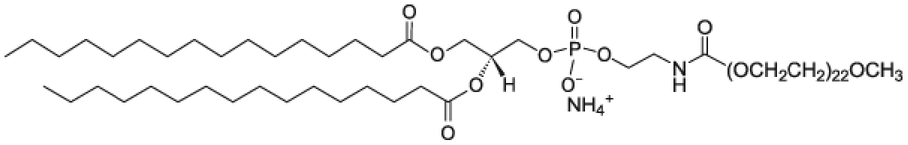	0.05-0.2
AMS	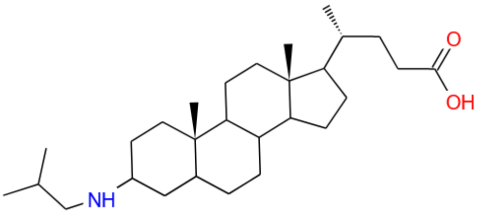	0.1***

*The molar ratio of the anionic CL^{2-} headgroups ν_{CL2-}=[1/2CL^{2-}]/([1/2CL^{2-}]+[DOPC])

** The molar ratio of the cationic lipid $DOTAP^{1+}$ $\nu_{DOTAP1+}$=[$DOTAP^{1+}$]/([$DOTAP^{1+}$]+[DOPC])

***The molar ratio of AMS ν_{AMS} =[AMS]/([AMS]+[DOPC]+[CL^{2-}])

Procedure S1. AMS synthesis.

Methyl 3β-(isobutylamino)-5β-cholan-24-oate was synthesized according:

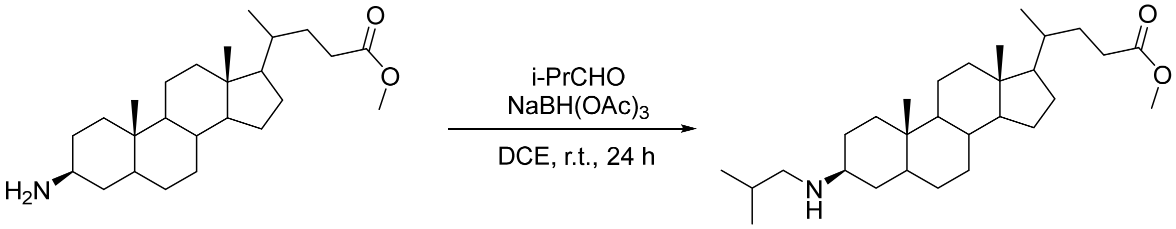

To a mixture of 97 mg (0.25 mmol) methyl 3β-amino-5β-cholan-24-oate and 25 μl (19.8 mg, 0.275 mmol) isobutyraldehyde in 6 ml of dry 1,2-dichloroethane 80 mg (0.375 mmol) of sodium triacetoxyborohydride was added at ambient temperature and obtained solution was stirred for 24 h at ambient temperature. The reaction mixture was diluted with CH_2Cl_2 (30 ml), washed twice with sat. aqueous solution of Na_2CO_3. Organic layer was separated, dried with Na_2SO_4 and solvents evaporated. The product was purified by column chromatography (CH_2Cl_2/MeOH 10:1). Yield – 91%, white powder. ^{1}H NMR (400 MHz, $CDCl_3$), δ: 3.66 (s, 3H, $COOCH_3$), 2.95 (bs, 1H, 3α-CH), 2.43 (d, *J* = 6.7 Hz, 2H, NCH_2), 2.39-2.17 (m, 2H, 23-CH_2), 1.99-1.75 (m, 6H), 1.67-1.02 (m, 21H), 0.96 (s, 3H, 19-CH_3), 0.93 (d, *J* = 6.7 Hz, 6H, ($\underline{CH}_3$)$_2$CH), 0.91 (d, *J* = 6.5 Hz, 3H, 21-CH_3), 0.64 (s, 3H, 18-CH_3).^{13}C NMR (100 MHz, $CDCl_3$): 174.7, 56.6, 55.9, 54.8, 52.9, 51.4, 42.7, 40.2, 40.0, 36.6, 35.6, 35.3, 35.1, 31.0, 30.3, 30.3, 28.2, 27.7, 26.9, 26.2, 24.1, 24.0, 23.8, 21.0, 20.9, 20.8, 18.2, 12.0.

3β-(Isobutylamino)-5β-cholan-24-oic acid (AMS) was synthesized according:

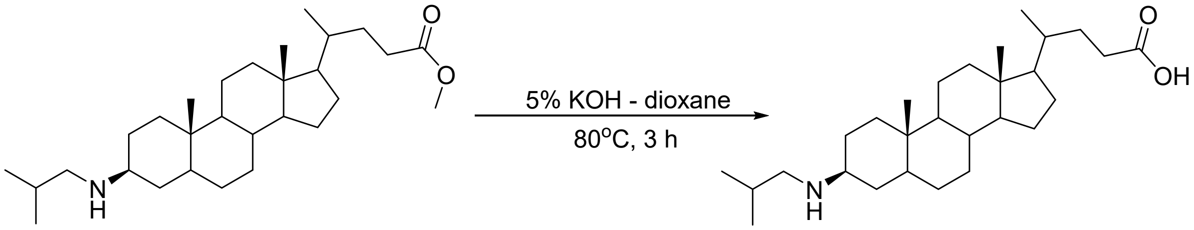

In a vial with a screw cap 59 mg (0,132 mmol) of methyl 3β-(isobutylamino)-5β-cholan-24-oate was placed and 5 ml of 1,4-dioxane with 5% aq. KOH (1:1) added. Obtained mixture was stirred for 3 h at 80°C, evaporated to the half of volume and acidified by HCl to pH 5-6. Product was extracted by EtOAc (3x30 ml), organic layer separated and dried with Na_2SO_4 and solvents were evaporated. Yield – 97%, white powder. ^{1}H NMR (400 MHz, CD_3OD), δ:3.46 (bs, 1H, 3α-CH), 2.86 (d, *J* = 6.9 Hz, 2H, N<u>CH</u>$_2$), 2.38-1.08 (m, 29H), 1.05 (d, *J* = 6.5 Hz, 6H, (<u>CH</u>$_3$)$_2$CH), 1.04 (s, 3H, 19-CH_3), 0.94 (d, *J* = 6.3 Hz, 3H, 21-CH_3), 0.70 (s, 3H, 18-CH_3). ^{13}C NMR (100 MHz, CD_3OD): 176.2, 57.7, 57.4, 57.0, 54.2, 43.9, 41.4, 41.3, 37.5, 36.9, 36.7, 36.0, 32.3, 32.1, 30.7, 29.2, 28.4, 27.4, 27.0, 26.7, 25.2, 23.6, 22.5, 22.1, 20.5, 18.8, 12.0. MALDI-TOF: 432.3966 $[M+H]^+$. Calculated for $C_{28}H_{50}NO_2$: 432.3836.

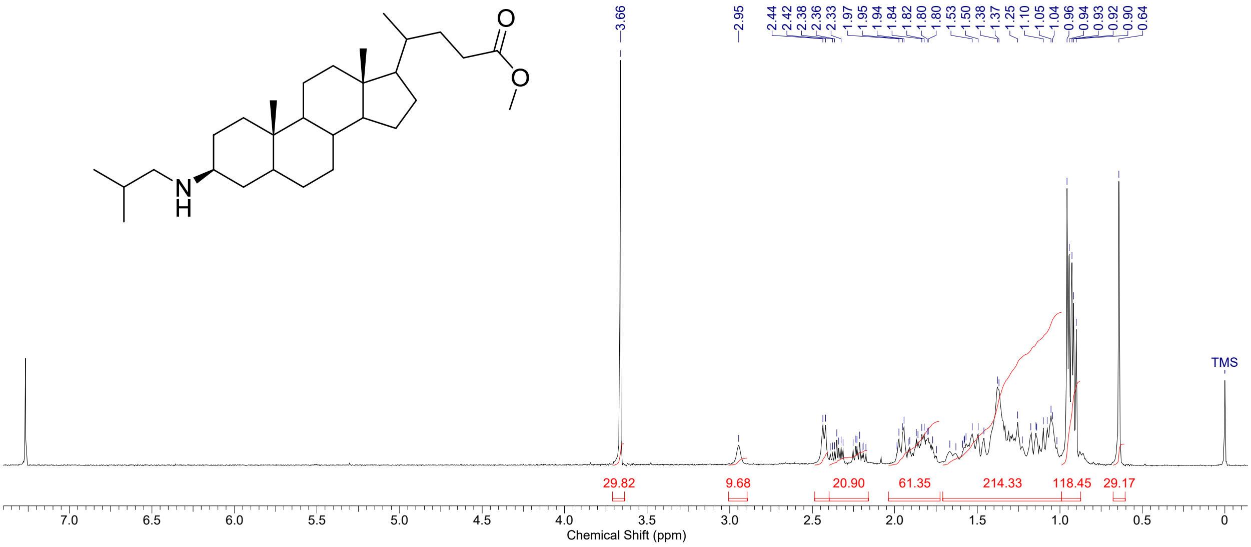

Figure S1. Methyl 3β-(isobutylamino)-5β-cholan-24-oate (^{1}H NMR, $CDCl_3$)

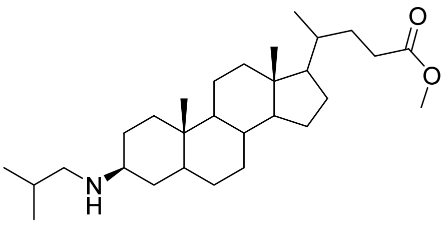

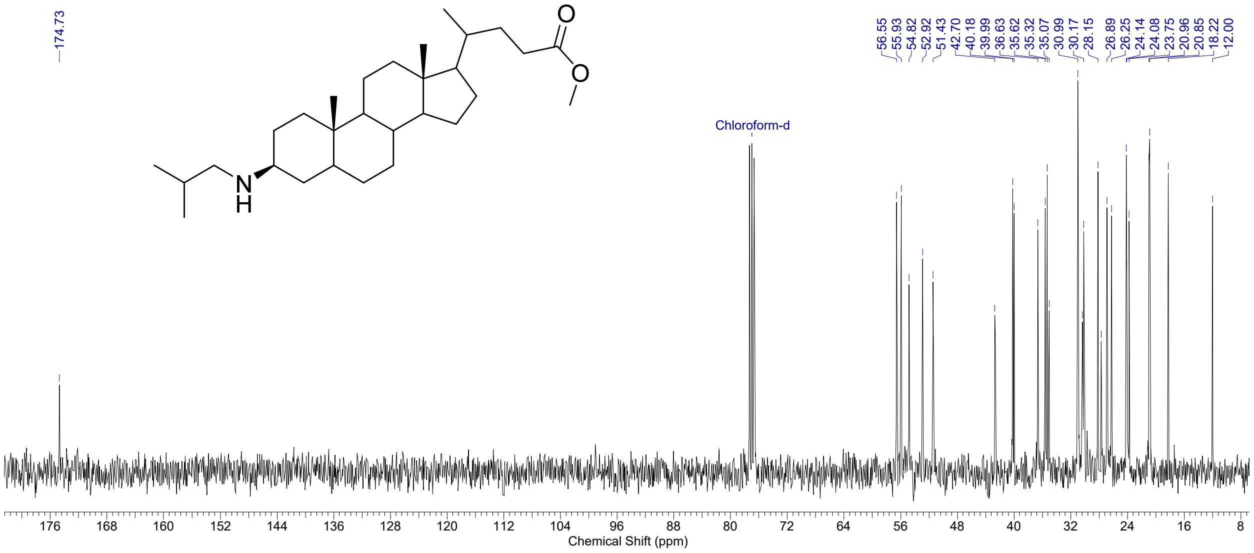

Figure S2. Methyl 3β-(isobutylamino)-5β-cholan-24-oate (^{13}C NMR, $CDCl_3$)

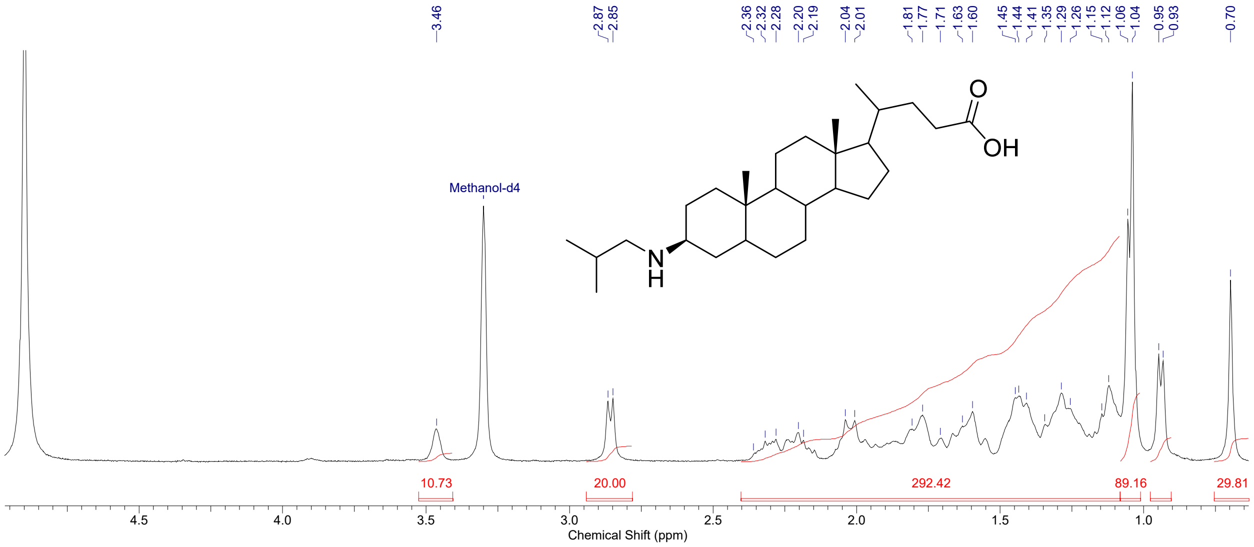

Figure S3. 3β-(Isobutylamino)-5β-cholan-24-oic acid (^1H NMR, CD_3OD) (AMS)

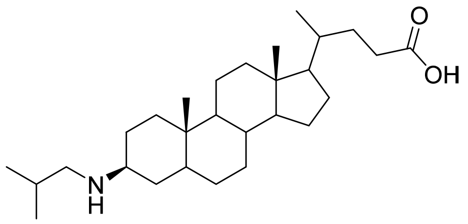

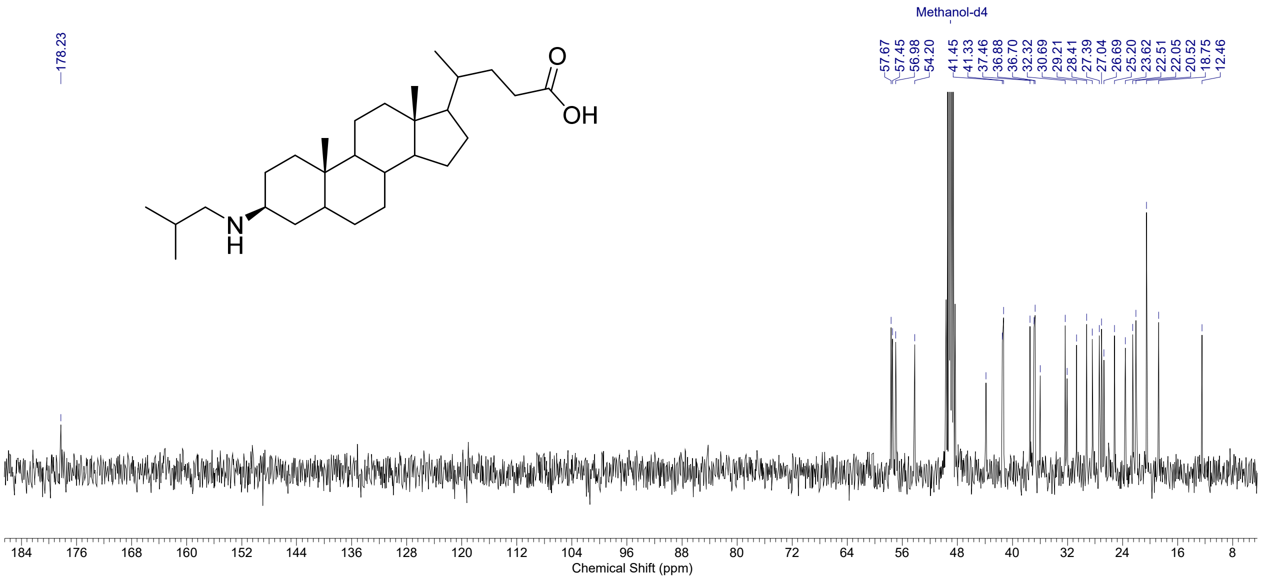

Figure S4. 3β-(Isobutylamino)-5β-cholan-24-oic acid (^{13}C NMR, CD_3OD) (AMS)

NMR spectra were recorded with a Bruker Avance 400 (1H 400 MHz, 13C 100.6 MHz) spectrometer at ambient temperature. Chemical shifts are presented in ppm (δ scale) and referenced to tetramethylsilane (δ=0 ppm) in the 1H NMR spectra and to the solvent signal in the 13C NMR spectra. MALDI-TOF spectra were recorded with a Bruker Daltonics UltraFlex instrument in a dithranol matrix using PEG 400 or PEG 600 as the internal standard.

a

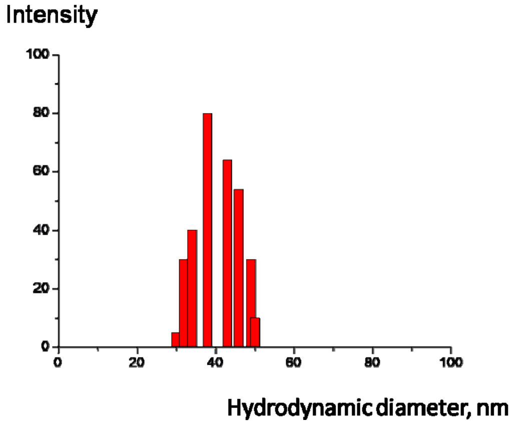

b

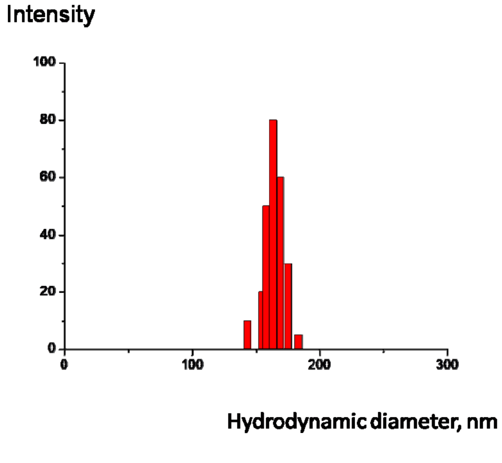

c

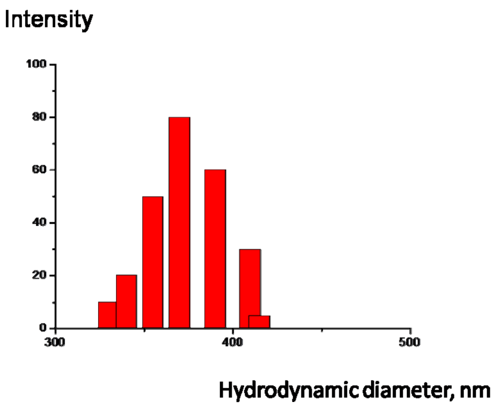

Figure S5. Size distribution of anionic CL^{2-}/DOPC/AMS (a) and cationic $DOTAP^{1+}$/DPPC/DPPE-PEG (b) vesicles and their saturated complexes (c). Molar ratio of DPPE-PEG equal to 0.1. Total liposome concentration: $C_{cationic}$=1 mg/ml, $C_{anionic}$ =0.4 mg/ml.

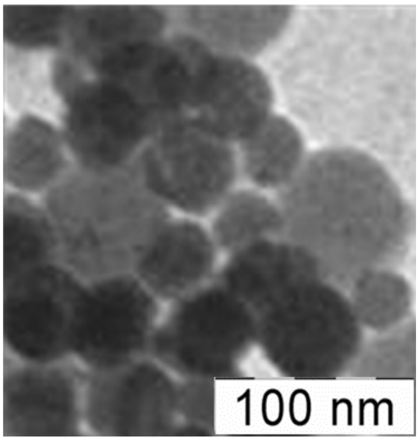

Figure S6. TEM image of complex of anionic CL^{2-}/DOPC/AMS vesicles with cationic $DOTAP^{1+}$/DPPC/DPPE-PEG vesicles. Molar ratio of DPPE-PEG equal to 0.1. The total liposome concentration: $C_{cationic}$=1 mg/ml, $C_{anionic}$ =0.4 mg/ml. 10^{-2} M TRIS buffer.

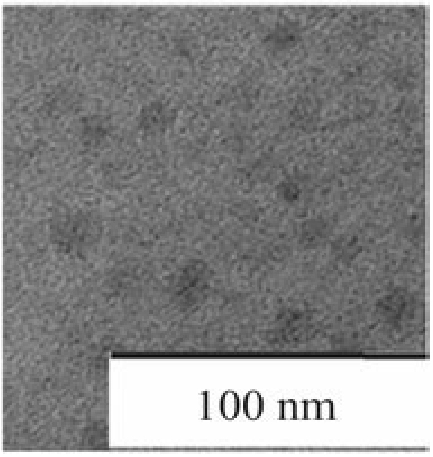

Figure S7. TEM image of the biodegradation products of multiliposomal complexes (MLC) in 72 h after addition of lipase. Anionic CL^{2-}/DOPC/AMS vesicles ; cationic $DOTAP^{1+}$/DPPC/DPPE-PEG vesicles , molar ratio of DPPE-PEG equal to 0.1. Saturated MLC were prepared at pH=8.0. Total lipid concentration used for liposome preparation: $C_{cationic}$=10 mg/ml, $C_{anionic}$ =4 mg/ml. Lipase concentration 5×10^{-1} mg/ml.

Procedure S2. Degree of binding of pH-sensitive anionic liposomes with the cationic

To determine whether all amount of pH-sensitive anionic liposomes binds to cationic the following experiment was carried out. Anionic liposomes with a fluorescent label embedded in the bilayer were obtained and complexed with cationic in different ratio. The obtained complexes were separated from the solution by centrifugation, and the fluorescence intensity of the label in the supernatant was detected. It was shown in a separate experiment that free liposomes did not precipitate under centrifugation. Analyzing the obtained results we found out that the quantitative binding of anionic liposomes was observed up to 0.6 mg/ml. Until this concentration all added pH-sensitive anionic liposomes were complexed with the cationic and no free anionic liposomes were found in the suspension.

Procedure S3. Evaluation of storage stability.

We conducted experiments to evaluate the storage stability of multiliposomal complexes at 4°C. It was estimated that at 4°C the multiliposomal complexes retain their size for at least 7 days. The data on the hydrodynamic diameter of the complexes are presented at Table S2.

Table S2. Hydrodynamic diameter of the complexes of anionic CL^{2-}/DOPC/AMS vesicles with cationic $DOTAP^{1+}$/DPPC/DPPE-PEG vesicles at 4°C

Hydrodynamic diameter, nm				
In 1 day	In 2days	In 3 days	In 6 days	In 7 days
325±12	310±23	320±20	330±16	318±18

Molar ratio of DPPE-PEG equal to 0.1. Total liposome concentration: $C_{cationic}$=1 mg/ml, $C_{anionic}$ =0.4 mg/ml.

Table S3

Hydrodynamic diameter of the multiliposomal complexes in 7 days of the incubation

.

Среда	20^0C	37^0C
Phosphate buffer (pH=7.5, 0,15-0,2 M NaCl)	350±20	340±20
Phosphate buffer (pH=7.0, 0,15-0,2 M NaCl)	340±15	320±10
RPMI 1640	320±30	330±40
DMEM/F12	350±15	360±20

Molar ratio of DPPE-PEG equal to 0.1. Total liposome concentration: $C_{cationic}$=1 mg/ml, $C_{anionic}$ =0.4 mg/ml.

Procedure S4. Evaluation of cytotoxicity.

Cytotoxicity of the liposomes towards human breast adenocarcinoma MCF-7 cells (Database name: ATCC, Accession Numbers:HTB22) was evaluated with a methyl-tetrazolium blue assay. The dye, penetrated into the living cells and attacked by redox enzymes, is reduced to formazan and precipitates as dark-blue crystals whereas in dead cells such transformation does not occur. The standard procedure involves incubation of the test suspension (solution) with the cells, addition of the dye solution, dissolution of formazan crystals in dimethylsulfoxide (DMSO), measurement of the optical density of the resulting solution and comparison of the result with the calibration curve in the absence of the tested objects. Briefly, the day before the experiment, MCF-7 cells were seeded on a 96-well plate (Biofil, China) at a density 3800 cells per well (in 0.1 mL DMEM/F12 (PanEco, Russia), 10% (v/v) fetal bovine serum (Hiclone, USA), 1% (v/v) L-glutamax (Sigma) and 1% (v/v) antibiotic solution (penicillin, streptomycin)(PanEco, Russia)). The next day, the culturing medium was removed and 0.1 mL of the solutions of liposomes at varying concentrations in the serum-free medium were placed in the wells for 1 h. In control wells (100% of surviving cells) complete medium was replaced with serum-free medium for 1 h. Then, the solutions were removed and the cells were cultured in 0.1 mL of complete medium for 72 h. The amount of living cells was assayed by addition of 0.1 mL of MTT solution (0.375 mg/mL) in the culturing medium for 4 h. Then, the medium was removed, violet crystals of formazan were dissolved in 0.1 mL of DMSO, and the optical density at 570 nm was measured on a VersaMax microplate reader (USA). The reference wavelength at 630 nm was used. The portion of survived cells was calculated as a ratio of optical density in well with certain concentration to that in control well. All runs were carried out in quadruplicates.
